# Pulmonary Artery Aneurysm in Behcet’s Disease Manifesting as Haemoptysis: A Case Report and Literature Review

**DOI:** 10.1093/omcr/omae097

**Published:** 2024-08-26

**Authors:** Ali Akeel Al-Yacopy, Ahmed Qasim Mohammed Alhatemi, Hashim Talib Hashim, Ahmed Dheyaa Al-Obaidi, Rand Abdulhussain

**Affiliations:** Fellow Iraqi Board of Cardiology, Coronary Care Unit, Al Nasiriyah Heart Hospital, Thi Qar, 64001, Iraq; Department of Internal Medicine, Al Nasiriyah Teaching Hospital, Thi Qar, 64001, Iraq; Head of Research Department, Warith Al Anbiyaa University, Karbala, 56001, Iraq; College of Medicine, University of Baghdad, Baghdad, 10011, Iraq; Pharmacy department, University of Huddersfield, Huddersfield, HD1 3DH, United Kingdom

**Keywords:** rheumatology, cardiology and cardiovascular systems, emergency medicine, immunology, radiology, nuclear medicine, and medical imaging

## Abstract

Pulmonary artery aneurysm (PAA) in Behcet’s disease is a rare yet potentially life-threatening manifestation, often presenting as haemoptysis. We present a case report of a 33-year-old male with recurrent haemoptysis and a history of oral and genital ulcers, diagnosed with Behcet’s disease complicated by PAA. Diagnostic workup, including imaging and laboratory tests, confirmed the diagnosis, leading to prompt initiation of immunosuppressive therapy and meticulous follow-up. The patient showed significant clinical improvement with reduced ulcer severity and no new episodes of haemoptysis during one-year follow-up. Our case underscores the importance of early recognition, multidisciplinary management, and close monitoring to optimize outcomes in Behcet’s disease with pulmonary involvement. A comprehensive literature review further elucidates the clinical nuances, diagnostic challenges, and therapeutic strategies associated with this rare manifestation, highlighting the need for collaborative care and tailored interventions to mitigate complications and improve patient prognosis.

Key clinical message

Behcet’s disease can present with pulmonary artery aneurysm, necessitating vigilant monitoring and prompt intervention to prevent severe complications like haemoptysis. Multidisciplinary collaboration is vital for tailored management and improved patient outcomes.

## Introduction

Pulmonary Artery Aneurysm (PAA) within the context of Behcet’s Disease represents a rare and potentially life-threatening manifestation that demands careful consideration [1]. Behcet’s Disease, a chronic autoimmune disorder characterized by recurrent oral and genital ulcers, skin lesions, and uveitis, can, in rare instances, involve the vascular system. When this inflammatory process extends to the pulmonary arteries, aneurysmal dilations may develop. Of particular concern is the manifestation of PAA leading to haemoptysis, a severe clinical event with implications for respiratory function and overall morbidity [2]. The relationship between Behcet’s Disease and PAA underscores the systemic nature of this vasculitis, requiring a comprehensive understanding for accurate diagnosis and timely intervention [3]. This introduction seeks to underscore the importance of recognizing PAA as a complication of Behcet’s Disease, emphasizing its potential to precipitate life-threatening haemoptysis [4]. As we delve into the discussion, we aim to explore the clinical nuances, diagnostic challenges, and management strategies associated with Pulmonary Artery Aneurysms in the context of Behcet’s Disease, shedding light on this intricate facet of vasculitic pathology [5].

## Case report

A 33-year-old male presented to the outpatient clinic with a one-month history of moderate haemoptysis, bright red in colour, associated with recurrent fever, chest infections, and shortness of breath. He has no past medical history and denies any substance abuse or chronic medication use. He is a non-smoker and non-alcoholic. There is no family history of autoimmune disease or premature deaths.

Upon detailed history-taking, it is revealed that he has had a history of persistent and recurrent oral ulcers for more than 10 years. Additionally, he experienced one episode of genital ulceration three years ago, and uveitis leading to blindness in the right eye occurred one year ago.

**Figure 1:**
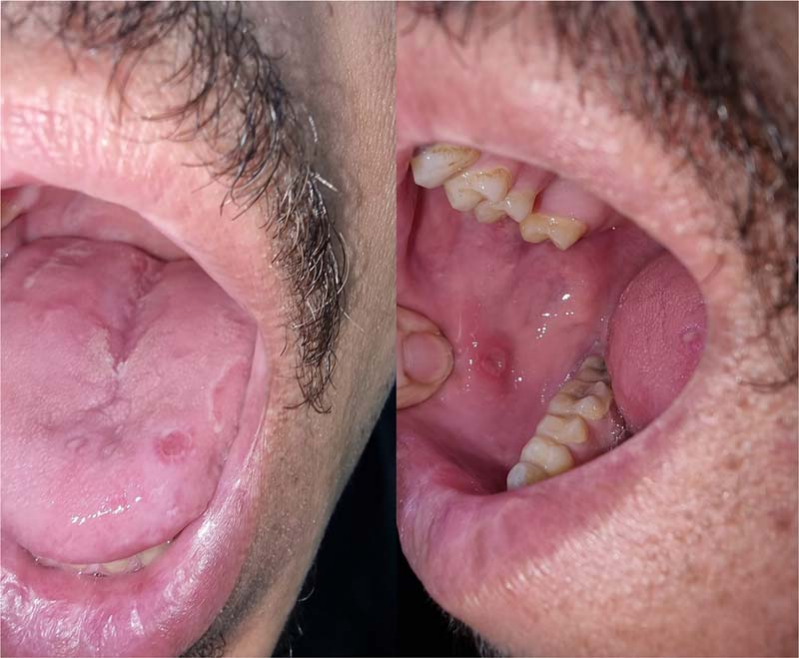
Oral aphthous ulcer on the buccal mucosa and the Tongue.

On examination, he is an average-built male in distress with pale conjunctiva and palmar creases. There are no signs of jaundice, lymphadenopathy, clubbing, or oedema. Respiratory, abdominal, and cardiac examinations were unremarkable. Ophthalmology examination of the right eye revealed phthisis bulbi (lost eye), while examination of the left eye showed a decrease in vision, cataract, and retinal vascular attenuation. Oropharyngeal examination revealed oral aphthous ulcer (Fig. 1).

No genital ulcer was observed at the time of examination. His vital signs were as follows: blood pressure 123/83 mmHg, respiratory rate 24 cycles/min, temperature 37.3 degrees Celsius, and oxygen saturation at 95% on room air.

**Figure 2:**
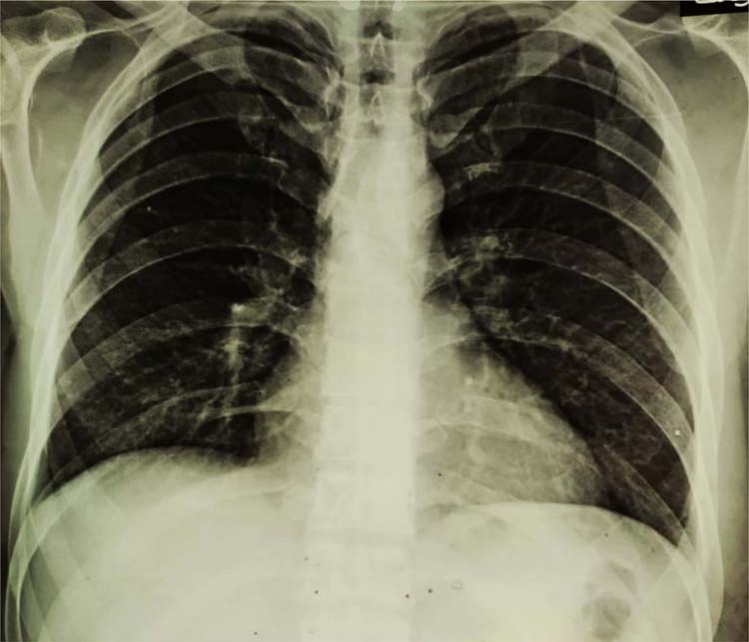
Chest X-ray PA view labelled as normal.

**Figure 3:**
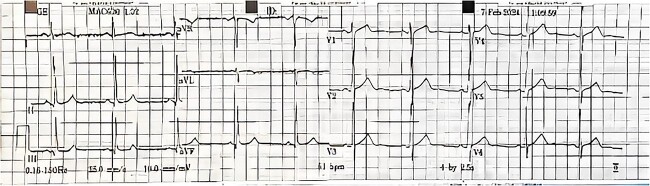
Initial ECG showing normal axis deviation sinus rhythm.

The top diagnosis considered as an emergency was pulmonary embolism, which needed to be excluded. We proceeded with a chest X-ray (Fig. 2) and an ECG (Fig. 3), both labelled as normal.

Chest infection was considered as one of the differential diagnoses. Given the high prevalence of tuberculosis in Iraq, three sputum samples were sent for Acid Fast Bacilli (AFB) test, all of which returned negative results. Blood samples were collected for a complete blood count, renal and liver function tests, inflammatory markers, and immune screening (Table 1).

**Table 1:** Initial blood tests showed highly elevated ESR and CRP, alongside anaemia and negative immune screening

Test	Results	Normal value
HB(g/dl)	10.3	12–16
WBC(10^3^/microliter)	9.7	4–11
PLT(10^3^/microliter)	377	250–450
Urea(mg/dl)	14	5–20
Creatinine(mg/dl)	0.9	0.7–1.3
ALT(U/l)	33	10–0
AST(U/l)	28	7–45
GGT(IU/l)	41	10–40
INR	1.1	1–1.5
PT(seconds)	12	11–13
aPTT(seconds)	22	30–40
CRP	79	<10
ESR	88	0–22
D-dimer	466	<500
ANA	1	6–10
C ANCA	2	11–50
P ANCA	1	11–50

His history, examination, and laboratory test results raised a high suspicion of autoimmune disease, specifically Behcet’s disease.

**Figure 4:**
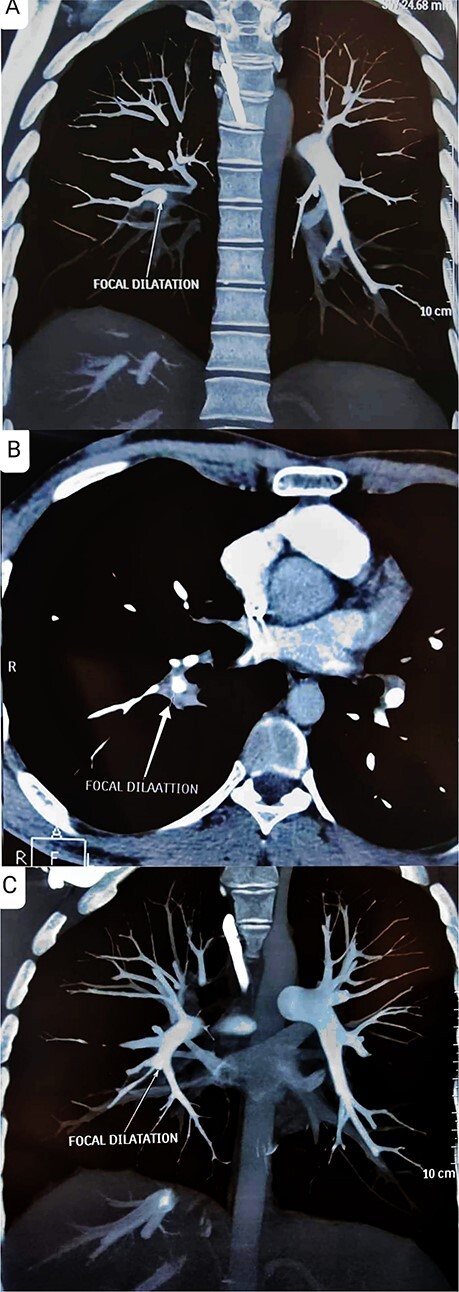
(A–C) CT pulmonary angiogram shows focal dilation of approximately 8 mm observed at the right lower lobe pulmonary artery, precisely at the first-order bifurcation (white arrows) and no fibrosis or parenchymal lung changes were detected.

We proceeded further with a pathergy test, and the result was the development of an inflammatory papule 4 cm in size within 24 h, which constitutes a positive result. Therefore, a computed tomography pulmonary angiography was ordered (Fig. 4A–C).

Echocardiography was ordered and revealed a normal ejection fraction without valvular abnormalities or regional wall motion anomalies, neither pericardial effusion.

His diagnosis was established as a case of Behcet’s disease complicated by pulmonary artery aneurysm according to the criteria published by the International Study Group for Behcet’s Disease.

We started him on intravenous methylprednisolone (1 g/day) for 3 days, then oral prednisone 60 mg once daily, which was subsequently tapered to 10 mg twice daily after one month. Intravenous cyclophosphamide at a dose of 1 g (15 mg/kg) was also given every 4 weeks for a total of 6 cycles, followed by oral azathioprine 100 mg once daily.

Regarding oral ulcers, we treated him with triamcinolone cream three times per day and colchicine 1 mg once daily for arthritis.

Two months following the initiation of treatment, the patient showed remarkable improvement in clinical symptoms and laboratory markers. Regular follow-ups were conducted to monitor his progress closely.

During the follow-up visits for one year, the patient reported a significant reduction in the frequency and severity of oral ulcers. The oral aphthous ulcer on the buccal mucosa and tongue showed signs of healing, with reduced inflammation and pain. Additionally, there were no new episodes of genital ulcers reported.

Laboratory investigations revealed a gradual normalization of inflammatory markers. The C-reactive protein (CRP) levels decreased steadily to 11. Similarly, the erythrocyte sedimentation rate (ESR) showed a downward trend at 9, reflecting decreased inflammatory activity in the body.

Imaging studies, including repeat chest X-rays and CT pulmonary angiography at 6 months after treatment, were performed to assess the status of the pulmonary artery aneurysm. Demonstrated stability in the size of the aneurysm, without any signs of progression or complications such as thrombosis or rupture.

Echocardiography examinations were conducted to monitor cardiac function and assess for any signs of cardiac involvement related to Behcet’s disease. Throughout the follow-up period, echocardiograms showed preserved ejection fraction and no evidence of valvular abnormalities or pericardial effusion.

The patient tolerated the treatment regimen well with no significant adverse effects reported. Regular monitoring of liver and renal function tests ensured the safety of medications such as cyclophosphamide and azathioprine.

**Figure 5:**
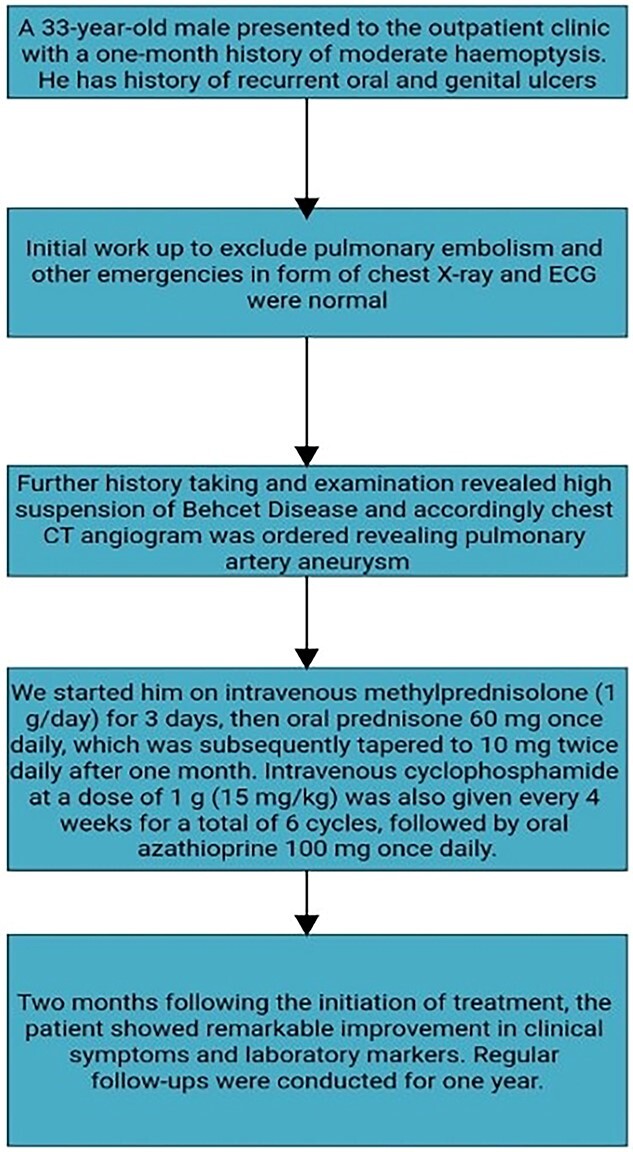
Summary of the patient presentation, work up and management.

Psychosocial support and counselling were provided to address any concerns or anxieties related to the chronic nature of Behcet’s disease and its treatment. The patient was encouraged to adhere to the prescribed medication regimen and advised on lifestyle modifications to optimize overall health and well-being. Figure 5 summarizes the overall investigation, management, and follow-up of the patient.

Overall, the multidisciplinary approach to management, combined with regular follow-up and monitoring, contributed to the successful stabilization of the patient’s Behcet’s disease and resolution of pulmonary artery aneurysm-related complications.

Table 2 provides a concise overview of reported cases of Behcet’s disease with pulmonary involvement. It highlights diverse clinical presentations, diagnostic approaches, and treatment strategies, emphasizing the multisystem nature of the condition. Cases exhibit varied manifestations, including haemoptysis, dyspnoea, oral ulcers, fever, and systemic inflammatory symptoms, highlighting the complexity of Behcet’s disease. Diagnostic modalities such as chest X-ray, CT imaging, echocardiography, and laboratory tests (CBC, CRP, ESR) are crucial for confirming the diagnosis and assessing disease severity. Management involves individualized approaches with immunosuppressive agents like corticosteroids, cyclophosphamide, and azathioprine. Surgical interventions may be necessary for severe complications. Long-term treatment aims for disease remission and prevention of relapses with maintenance immunosuppressive therapy, close monitoring, and regular follow-up. Behcet’s disease with pulmonary involvement presents diagnostic and therapeutic challenges. Early recognition and multidisciplinary management are essential for improving outcomes and minimizing complications.

**Table 2:** A summary of reported cases of Behcet’s disease presenting with pulmonary aneurysm and their management

Reported cases	Patients’ relevant demographics	Clinical features	Investigations	Treatment plan
Kasikcioglu et al. [9]	32 year old male with no past medical history	Bilateral ankle oedema, abdominal discomfort, and haemoptysis	Chest X-ray and helical computed tomography (CT) scan	Colchicine and cyclophosphamide for 24 months
Kage et al [10]	42 year old male with no past medical history	Recurrent oral ulcers and intermittent haemoptysis	Complete blood count and chest compund tomography	Methylprednisolone, cyclophosphamide and azathioprine
Abd Elrazak and Al-Dalaan [11]	Case 1: 14-year-old Saudi female Case2: 22-year-old Saudi male	Recurrent oral ulcers, fever and headaches recurrent oral and genital ulcers, uveitis, erythema nodosum and a meningitislike picture	CBC, chest X-ray and CT chest CBC, immune screening, chest X-ray and pulmonary angiogram	Prednisone and chlorambucil Prednisolone, azathioprine and colchicine
Uh et al. [12]	21-year-old Korean woman	dyspnoea and cough for the past 3 months	Chest X-ray, CT chest and pulmonary angiogram	Prednisolone and lobectomy
Alagha et al. [1]	31-year-old female without past medical history	Sudden onset of frank haemoptysis of one-day duration, which was large in amount (roughly 150 ml) and associated with shortness of breath	chest CT angiography	Methylprednisolone, cyclophosphamide and prednisolone
Law et al. [13]	45-year-old woman with a history of Behçet’s disease with pulmonary involvement including PAAs, factor V Leiden, and prior cerebral venous thrombosis and pulmonary embolism	massive hemoptysis	Chest X-ray,computed tomography angiography and bronchoscopy	Right upper lobectomy
Roguin et al. [14]	26-year-old Behçet’s disease patient with large right atrial thrombus, pulmonary aneurysms and aortic pseudoaneurysm that developed 17 years after surgery for bilateral renal artery stenosis	fever of unknown origin associated with chest pain, dyspnea, cough and haemoptysis	Chest X-ray, pulmonary CT, open lung biopsy and echocardiogram	Open heart surgery
Kotecha et al. [15]	36-year-old male with a history of anterior uveitis and mild bronchiectasis	2-month history of worsening dyspnoea, weight loss, haemoptysis, oral ulceration, erythema nodosum and superficial thrombophlebitis	CRP, ESR, ANA and computed tomography pulmonary angiogram (CTPA)	Methylprednisolone, cyclophosphamide, azathioprine, prednisolone and infliximab

## Discussion

Behcet’s Disease, a multisystemic vasculitis, can exceptionally manifest as a Pulmonary Artery Aneurysm (PAA), with potentially severe consequences, notably haemoptysis [4]. The immune-mediated inflammation in Behcet’s Disease extends to blood vessels, affecting arteries and veins of varying sizes. When this inflammation involves the pulmonary arteries, it may lead to the development of an aneurysm, a dilation of the vessel wall that can be particularly precarious [6].

Haemoptysis, the expectoration of blood from the respiratory tract, arises when the PAA ruptures or leaks, posing a life-threatening complication [6]. This manifestation demands heightened clinical awareness due to its rarity and potential for rapid deterioration. The challenge lies in the fact that Behcet’s Disease can mimic other pulmonary conditions, delaying accurate diagnosis [5].

Early detection is paramount. A pulmonary angiogram is the gold standard for diagnosing pulmonary aneurysms. However, it may be risky in Behcet disease patients as the venous puncture or rapid injection of contrast may initiate or worsen an existing thrombus. Additionally, a pulmonary angiogram may not detect thrombosed pulmonary aneurysms. Thus, helical computed tomography angiography (CTA) has emerged as a non-invasive and excellent way of detecting pulmonary aneurysms in Behcet disease. Magnetic resonance imaging (MRI) could be an alternative option for patients who cannot get a CTA, such as those with impaired renal function or severe iodine allergy [7].

Management varies based on organ system involvement, severity, age, and gender. Colchicine serves as the cornerstone for mucocutaneous lesions, while other manifestations may necessitate agents like cyclosporine and anti-TNF agents. Vascular involvement, attributed to endothelial inflammation and thrombosis, underscores the importance of reducing inflammation. Though the role of anticoagulation is debated, immunosuppressive agents such as corticosteroids, azathioprine, and cyclosporine A show promise in preventing thrombotic events [8].

Limited evidence exists for treating pulmonary artery aneurysms in Behçet’s, but immunosuppressive approaches demonstrate efficacy. Intravenous corticosteroids and cyclophosphamide followed by oral corticosteroids with other immunosuppressive agents have resulted in improved survival rates [8].

Given the potential fatal consequences of PAA rupture, patient education is imperative. Recognizing symptoms like recurrent haemoptysis, chest pain, or dyspnea is essential for early medical intervention. Furthermore, support groups and mental health resources are valuable, acknowledging the emotional toll of living with a rare, potentially life-threatening condition [6].

## Conclusion

Pulmonary artery aneurysm (PAA) in Behcet’s disease presents a complex clinical scenario with potentially severe consequences, such as haemoptysis. Our case report and literature review stresses the importance of recognizing this rare manifestation early and implementing a multidisciplinary approach for optimal management. Through timely diagnosis, individualized treatment strategies, and meticulous follow-up, we can effectively mitigate complications and improve patient outcomes. Continued research and collaborative efforts are essential to further enhance our understanding of this rare but significant aspect of Behcet’s disease, ultimately improving patient care and prognosis.

## Consent

Written consent form for publication of his details was obtained from the patient.

## Guarantor

Ahmed Qasim Mohammed Alhatemi.

## Data Availability

The data that support the findings of this study are available from the corresponding author upon reasonable request.
